# Polyphenol-Rich *Aronia melanocarpa* Juice Consumption Affects *LINE-1* DNA Methylation in Peripheral Blood Leukocytes in Dyslipidemic Women

**DOI:** 10.3389/fnut.2021.689055

**Published:** 2021-06-17

**Authors:** Ljiljana Stojković, Manja Zec, Maja Zivkovic, Maja Bundalo, Maja Bošković, Marija Glibetić, Aleksandra Stankovic

**Affiliations:** ^1^Laboratory for Radiobiology and Molecular Genetics, Department of Health and Environmental Research, “Vinča” Institute of Nuclear Sciences—National Institute of the Republic of Serbia, University of Belgrade, Belgrade, Serbia; ^2^Centre of Research Excellence in Nutrition and Metabolism, Institute for Medical Research—National Institute of the Republic of Serbia, University of Belgrade, Belgrade, Serbia; ^3^Department of Nutritional Sciences, University of Arizona, Tucson, AZ, United States; ^4^Institute of Experimental Biomedicine, University Hospital Würzburg, Würzburg, Germany

**Keywords:** *Aronia melanocarpa*, polyphenols, polyunsaturated fatty acids, *LINE-1*, methylation, peripheral blood leukocytes, cardiovascular risk

## Abstract

Cardiovascular disease (CVD) is associated with alterations in DNA methylation and polyunsaturated fatty acid (PUFA) profile, both modulated by dietary polyphenols. The present parallel, placebo-controlled study (part of the original clinical study registered as NCT02800967 at www.clinicaltrials.gov) aimed to determine the impact of 4-week daily consumption of polyphenol-rich *Aronia melanocarpa* juice (AMJ) treatment on *Long Interspersed Nucleotide Element-1* (*LINE-1*) methylation in peripheral blood leukocytes and on plasma PUFAs, in subjects (*n* = 54, age range of 40.2 ± 6.7 years) at moderate CVD risk, including an increased body mass index, central obesity, high normal blood pressure, and/or dyslipidemia. The goal was also to examine whether factors known to affect DNA methylation (folate intake levels, *MTHFR* C677T gene variant, anthropometric and metabolic parameters) modulated the *LINE-1* methylation levels upon the consumption of polyphenol-rich aronia juice. Experimental analysis of *LINE-1* methylation was done by MethyLight method. *MTHFR* C677T genotypes were determined by the polymerase chain reaction–restriction fragment length polymorphism method, and folate intake was assessed by processing the data from the food frequency questionnaire. PUFAs were measured by gas–liquid chromatography, and serum lipid profile was determined by using Roche Diagnostics kits. The statistical analyses were performed using Statistica software package. In the comparison after vs. before the treatment period, in dyslipidemic women (*n* = 22), we observed significant decreases in *LINE-1* methylation levels (97.54 ± 1.50 vs. 98.39 ± 0.86%, respectively; *P* = 0.01) and arachidonic acid/eicosapentaenoic acid ratio [29.17 ± 15.21 vs. 38.42 (25.96–89.58), respectively; *P* = 0.02]. The change (after vs. before treatment) in *LINE-1* methylation directly correlated with the presence of *MTHFR* 677T allele, average daily folate intake, and the change in serum low-density lipoprotein cholesterol but inversely correlated with the change in serum triacylglycerols (*R* = 0.72, *R*^2^ = 0.52, adjusted *R*^2^ = 0.36, *P* = 0.03). The current results imply potential cardioprotective effects of habitual polyphenol-rich aronia juice consumption achieved through the modifications of DNA methylation pattern and PUFAs in subjects at CVD risk, which should be further confirmed. Hence, the precision nutrition-driven modulations of both DNA methylation and PUFA profile may become targets for new approaches in the prevention of CVD.

## Introduction

Pathogenesis of human chronic diseases, such as cancer and cardiovascular disease (CVD), is related to aberrant global and locus-specific DNA methylation patterns (1, 2). Methylation of DNA, catalyzed by DNA methyltransferases (DNMTs), is one of the main epigenetic processes, which most commonly occurs at cytosine-guanine (CpG) dinucleotide clusters and results in downregulation of gene expression (3).

Various exogenous and endogenous factors modulate DNA methylation. Folate (vitamin B9) is found in a variety of plant foods and participates in one-carbon metabolism, resulting in the formation of S-adenosyl-methionine (SAM) that acts as a methyl group donor (4). Methylenetetrahydrofolate reductase (MTHFR) activation catalyzes the conversion of homocysteine to methionine, which is a direct precursor of SAM. The presence of T allele at a common C677T (Ala222Val) *MTHFR* gene polymorphic site is associated with a decreased activity of the enzyme, thus reducing the methyl group bioavailability and subsequently inhibiting the methylation of DNA (5).

Methylation of *Long Interspersed Nucleotide Element-1* (*LINE-1*), the largest member of the *LINE* retrotransposon family of DNA repeat elements (comprising about 17% of the human genome) (6), is considered a surrogate marker of global DNA methylation (7, 8). Methylation status of *LINE-1* in peripheral blood leukocytes is associated with metabolic parameters, such as blood glucose and lipid profiles (9, 10), and global DNA methylation in these cells has been found to depend on demographic and lifestyle factors: age, gender, blood pressure, body mass index (BMI), and dietary habits, including polyunsaturated fatty acid (PUFA) supplementation (9, 11–14). In addition, the studies have reported that the inhibition of DNA methylation may prevent the progression of cancer and CVD (1, 2, 15). Hence, the methylation status of *LINE-1* in peripheral blood leukocytes represents a potential CVD biomarker, and certain lifestyle factors, like diet, may modulate cardiovascular risk by influencing alterations in DNA methylation patterns, thus emphasizing the importance of precision nutrition strategies in the prevention of CVD.

Regular consumption of *Aronia melanocarpa* juice is associated with CVD beneficial effects in human studies (16–18) and animal models (19). Aronia is rich in bioactive polyphenols (20) and, compared with other berry fruits, contains higher levels of polyphenolic compounds (21). Of note, dietary polyphenols are reported to favorably modulate DNA methylation status by inhibiting DNMT activity (22). In addition, aronia polyphenols affected the composition of plasma PUFAs in individuals at CVD risk (23). To the best of our knowledge, no study has examined the relation between polyphenols contained in aronia berry and DNA methylation status. Therefore, the present parallel, placebo-controlled, 4-week study aimed to investigate the interconnection between daily consumption of polyphenol-rich aronia juice and *LINE-1* methylation in peripheral blood leukocytes and plasma fatty acids, in subjects at moderate CVD risk. We also examined whether folate intake and *MTHFR* C677T gene variant, as well as the anthropometric and metabolic parameters, modulated the *LINE-1* methylation levels upon the consumption of polyphenol-rich aronia juice.

## Materials and Methods

### Study Design, Study Subjects, and Intervention Treatments

The current research represents a parallel, placebo-controlled, 4-week nutritional intervention. The research is designed as a substudy, forming part of the original clinical study that lasted 6 months, and is registered at ClinicalTrials.gov as NCT02800967. The original study included nonsmoking adults at moderate CVD risk, defined as the presence of at least one of the following: increased BMI (25–30 kg/m^2^), central obesity (waist circumference ≥ 80 cm for women and ≥ 94 cm for men), and high normal blood pressure [systolic/diastolic blood pressure (SBP/DBP) > 120/80, ≤ 139/89 mm Hg]. Exclusion criteria were the presence of chronic disease, self-reported allergy to polyphenols, pregnancy, lactation, blood donation 16 weeks before the start of the study, and parallel participation in another clinical trial. During the course of the study, the participants were asked to follow their habitual diet, including study treatments as part of it, and to do their usual physical activity. They were also asked to strictly refrain from berries and berry products and to avoid excess amounts of polyphenol-rich food, inclusive of olive oil, green tea, and nuts.

**Figure 1:**
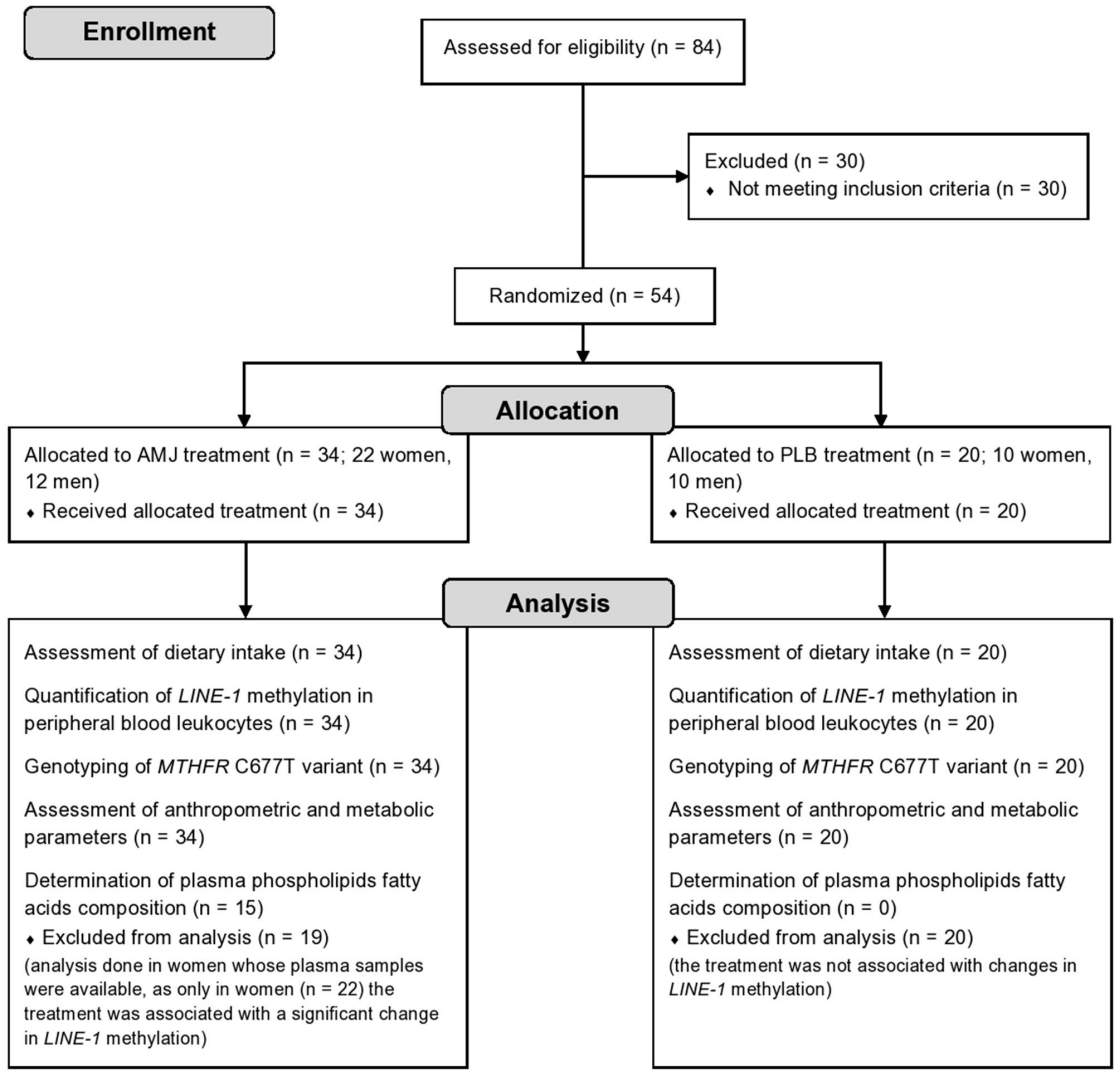
Study flow diagram. *N*, number of subjects; AMJ, polyphenol-rich *Aronia melanocarpa* juice treatment; PLB, polyphenol-free beverage; placebo, treatment.

For purposes of the current substudy, additional *a posteriori* inclusion criterion was the presence of dyslipidemia defined as either elevated serum total cholesterol (≥5.2 mmol/l), elevated low-density lipoprotein cholesterol (LDL-C) (≥3.4 mmol/l), or elevated serum triacylglycerols (≥1.7 mmol/l). In order to address the objectives of the substudy, 54 subjects were included, whose *LINE-1* methylation status in peripheral blood leukocytes and *MTHFR* C677T gene variant were additionally analyzed. They received either original polyphenol-rich *A. melanocarpa* juice (assigned as AMJ treatment, *N* = 34 subjects) or polyphenol-free placebo beverage (assigned as PLB treatment, *N* = 20 subjects). The research flow diagram is shown in Figure 1.

The original polyphenol-rich aronia juice used in the study was registered at the Serbian Ministry of Health as a dietary supplement and was donated from “Nutrika” LTD (Belgrade, Serbia). The placebo drink was made to match the appearance, taste, and nutritional composition of the original aronia juice, but without bioactive polyphenols. It was previously reported that the daily amount of 100 ml of the placebo was safe for human consumption (24). Total polyphenols in the original aronia juice were determined using a modified Folin-Ciocalteu method (25), and it was found that the consumed daily amount of 100 ml of the juice contained 1,177.11 mg of gallic acid equivalents of polyphenols. Proanthocyanidins were highly represented among the contained polyphenols, as demonstrated in the analysis of the composition of herein used aronia juice (24). The study compliance was assessed according to returned empty bottles of intervention drinks and self-reports.

The study protocol adhered to the regulations of the 1975 Declaration of Helsinki and was approved by Clinical Hospital Centre Zemun, Belgrade, Serbia, Ethics Committee Approval, No: 2125, 2013. The written informed consent was given by all participants before the commencement of the study.

### Sample Collection

Study participants were instructed for overnight fasting, and venous blood was collected the next morning between 8 and 9 AM into the sample tubes for serum and ethylenediaminetetraacetic acid (EDTA)-evacuated tubes. The sample collection was done at two time points, before and after the corresponding 4-week treatments (AMJ and PLB).

### Assessment of Study Variables

For the assessment of baseline dietary intake, trained staff conducted structured interviews with study subjects and collected data by using the food frequency questionnaire and repeated 24-h dietary recalls. The subjects were assisted with 125-item photo-booklet containing simple foods and composite dishes (26). Data from dietary recalls were analyzed using the nutritional platform for comprehensive diet evaluation (26, 27).

Bio-impedance analyzer TANITA UM072 balance (TANITA Health Equipment H.K. Ltd, Hong Kong, China) was used for the determination of body weight. Total cholesterol, high-density lipoprotein cholesterol (HDL-C), LDL-C, triacylglycerols, and glucose from serum were determined by Roche Diagnostics Kits, using the chemistry analyzer (Cobas c111, Roche Diagnostics, Basel, Switzerland).

The procedure of determination of plasma phospholipid fatty acids composition was previously described in detail by Pokimica et al. (23). Briefly, plasma lipids were extracted using a 2:1 chloroform–methanol mixture with 2,6-di-tert-butyl-4-methylphenol (10 mg/100 ml) added as an antioxidant. Phospholipids were separated from other lipid subclasses on a thin-layer chromatography silica plate using a mixture of petroleum ether, diethyl ether, and acetic acid (87:12:1). The methyl esters of fatty acids were obtained by transmethylation with 2M sodium hydroxide in methanol, at 85°C for 1 h, and with 1M sulfuric acid in methanol, at 85°C for 2 h. The mixture was cooled down to room temperature and centrifuged at 1,860 × g for 15 min, and the upper phase was dried up using a stream of nitrogen. The fatty acid methyl esters were recovered in hexane and separated by using RTX 2330 capillary column (60 m × 0.25 mm × 0.2 μm; Restek, Bellefonte, PA, United States), on Shimadzu GC-2014 gas chromatograph (Kyoto, Japan) with flame ionization detector. The flow of air, hydrogen, and helium (carrier gas) was 320, 30, and 5 ml/min, respectively. The temperature of the detector was 260°C and of the injection port 220°C. The initial column temperature of 140°C was maintained for 5 min and then increased to 220°C at a rate of 3°C/min, and 220°C was kept for 20 min. Fatty acids were identified by comparing peak retention times with calibration mixtures (PUFA-2, Supelco, Bellefonte, PA, United States, and 37 FAMEs mix, Sigma Chemical Co., St. Louis, MO, United States). The amounts of individual fatty acids in plasma phospholipids were presented as the relative area percentage of the total pool of detected fatty acids.

### Analysis of *LINE-1* Methylation

The fasting peripheral blood samples of each participant, collected with EDTA before and after the corresponding treatments, were used for the total leukocyte genomic DNA isolation, by a phenol–chloroform extraction-based method (28). The quantity of DNA was estimated with BioSpecnano spectrophotometer (Shimadzu Biotech, Kyoto, Japan).

Five hundred (500) ng of each sample genomic DNA was used for sodium bisulfite conversion with EpiTect® Bisulfite kit (Qiagen, Hilden, Germany). Bisulfite-converted DNA was then used for the quantification of *LINE-1* methylation by MethyLight real-time PCR. To normalize DNA input, an Alu sequence-based real-time PCR control reaction was performed in parallel with each *LINE-1* reaction. The method was previously developed and validated, its precision and reproducibility were confirmed (8, 29), and the currently used protocol was described in detail by Božović et al. (29).

### *MTHFR* Genotyping

The genotypes of *MTHFR* C677T variant were determined from the total leukocyte genomic DNA samples, by PCR–restriction fragment length polymorphism method reported in Coppedè et al. (30), in all subjects included in the methylation analysis.

### Statistical Analyses

The comparisons of *MTHFR* C677T genotype frequencies between groups and estimation of deviations from Hardy–Weinberg equilibrium were done with Fisher's exact test and the χ^2^ test. Due to a small number of the TT genotype carriers (*N* <3) in at least one of the analyzed groups, we applied the dominant genotype model, CT + TT vs. CC, instead of the additive. Depending on the distribution of continuous variables (age, average daily energy intake, average daily folate intake, SBP, DBP, waist circumference, BMI, serum glucose, triacylglycerols, total cholesterol, HDL-C, LDL-C, plasma fatty acids, and *LINE-1* methylation levels), tested by the Shapiro–Wilks test, the appropriate parametric or non-parametric tests were performed. Between-group comparisons were done using a *t*-test or the Mann–Whitney U-test, while within-group comparisons (after vs. before each treatment) were done by *t*-test for dependent samples or the Wilcoxon matched-pairs test, for normally and non-normally distributed data, respectively. The treatment effects were investigated in the complete sample and in women and men, separately. The relationship between *LINE-1* methylation and the anthropometric and metabolic parameters (age, average daily folate intake, average daily energy intake, SBP, DBP, waist circumference, BMI, serum glucose, triacylglycerols, total cholesterol, HDL-C, LDL-C, and plasma fatty acids) was investigated by regression analyses. T-test/Mann–Whitney *U*-test was used to examine the association between *LINE-1* methylation and *MTHFR* genotypes, by a genotype model, CT+TT vs. CC. In the statistical tests, *P* < 0.05 were considered statistically significant. The statistical analyses were performed using Statistica 8.0 software package (StatSoft, Inc. 1984-2007).

## Results

### Baseline Characteristics of the Study Subjects and Effects of Polyphenol-Rich Aronia Juice Consumption on Cardiometabolic Parameters

Baseline characteristics, determined before each of the two treatments, with polyphenol-rich aronia juice (AMJ) and polyphenol-free beverage (placebo, PLB), for the whole treated study groups are shown in Table 1. For the treated groups separated by gender, the baseline characteristics are shown in Supplementary Table 1. There were no significant differences in the baseline parameters (AMJ vs. PLB) within any of the groups (whole sample, women and men), except for serum total cholesterol in men [AMJ vs. PLB = 5.9 ± 1.0 vs. 4.9 ± 1.2 mmol/l; P (*t*-test) = 0.04] (Table 1 and Supplementary Table 1).

**Table 1:** Baseline characteristics of the study participants.

	**AMJ**	**PLB**	***P***
No. of subjects	34	20	
Age (years)	41.1 ± 6.6	38.5 ± 6.8	0.17
Average daily energy intake (kCal)	2075 ± 555	1745 (1168–3385)^#^	0.29
Average daily folate intake (μg)	239.7 ± 75.2	203.3 (98.2–461.5)^#^	0.54
SBP (mmHg)	118.3 ± 13.4	120.6 ± 16.2	0.57
DBP (mmHg)	73.2 ± 10.0	74.0 ± 13.7	0.82
Waist circumference (cm)	89.8 ± 10.9	92.2 ± 15.7	0.51
BMI (kg/m^2^)	27.4 ± 3.5	27.8 ± 6.2	0.78
Glucose (mmol/l)	4.8 (3.8–7.2)^#^	5.1 ± 0.8	0.27
TAG (mmol/l)	0.9 (0.4–4.1)^#^	0.9 (0.5–5.0)^#^	0.72
TC (mmol/l)	5.5 ± 1.1	5.2 ± 1.0	0.27
HDL-C (mmol/l)	1.5 (0.8–2.9) ^#^	1.6 ± 0.4	0.54
LDL-C (mmol/l)	3.5 ± 0.9	3.3 ± 1.0	0.56
*******MTHFR*** **C677T genotype, No. (%)**			
CC	15 (44.1)	7 (35.0)	
CT + TT	19 (55.9)	13 (65.0)	0.58

*Continuous variables with a normal distribution are presented as mean ± standard deviation;*

# *continuous variables with a non-normal distribution are presented as median (minimum–maximum); P-values related to the between-treatment difference in the variable distribution, significant difference at P < 0.05.*

*The observed MTHFR C677T genotype frequencies are consistent with Hardy–Weinberg equilibrium, in each analyzed group (χ^2^ test, P > 0.05).*

*AMJ, polyphenol-rich Aronia melanocarpa juice treatment; PLB, polyphenol-free beverage, placebo treatment; SBP, systolic blood pressure; DBP, diastolic blood pressure; BMI, body mass index; TAG, triacylglycerols; TC, total cholesterol; HDL-C, high-density lipoprotein cholesterol; LDL-C, low-density lipoprotein cholesterol*.

Within-group comparisons of treatment effects (after vs. before treatment) toward anthropometric and metabolic parameters are presented in Supplementary Table 2. Even though significant differences were found for DBP, waist circumference, glucose, and HDL-C [P (*t*-test for dependent samples/Wilcoxon matched pairs test) <0.05] (Supplementary Table 2), only values for waist circumference dropped out of the clinical ranges.

### Effects of Polyphenol-Rich Aronia Juice Consumption on *LINE-1* Methylation

**Figure 2:**
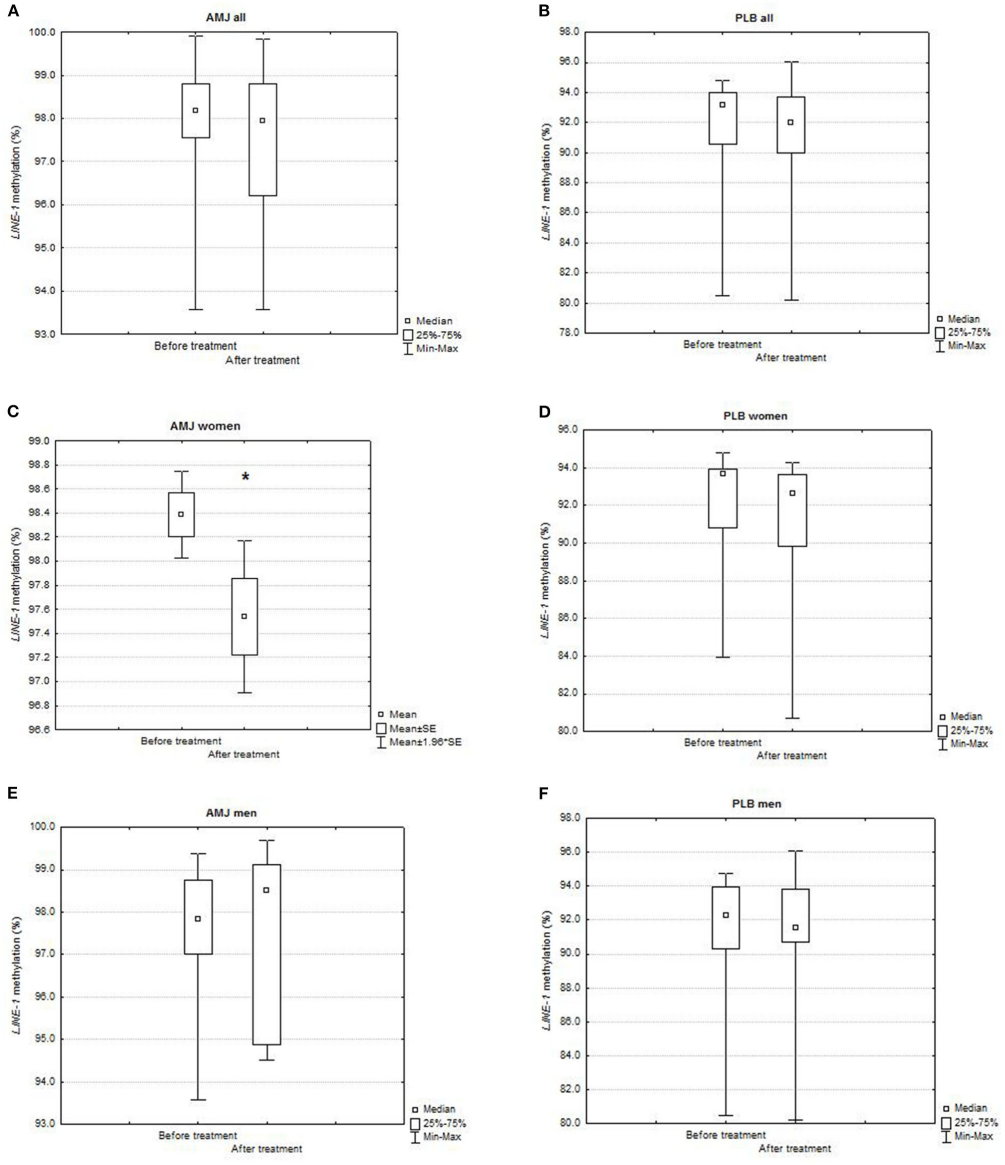
Comparison of *LINE-1* methylation levels (%) before and after treatment in: **(A)** all subjects on AMJ treatment (*N* = 34; Wilcoxon matched pairs test, *P* = 0.14), **(B)** all subjects on PLB treatment (*N* = 20; Wilcoxon matched pairs test, *P* = 0.10), **(C)** women on AMJ treatment (*N* = 22; *t*-test for dependent samples, *P* = 0.01), **(D)** women on PLB treatment (*N* = 10; Wilcoxon matched pairs test, *P* = 0.11), **(E)** men on AMJ treatment (*N* = 12; Wilcoxon matched pairs test, *P* = 0.53), **(F)** men on PLB treatment (*N* = 10; Wilcoxon matched pairs test, *P* = 0.65). AMJ, polyphenol-rich *Aronia melanocarpa* juice treatment; PLB, polyphenol-free beverage, placebo treatment; mean represents normally distributed *LINE-1* methylation levels (%); median represents non-normally distributed *LINE-1* methylation levels (%); SE, standard error; statistical significance at *P* < 0.05 (^*^); *N*, number of subjects.

The comparisons of *LINE-1* methylation levels after vs. before polyphenol-rich AMJ treatment and polyphenol-free beverage (placebo) treatment (PLB) are presented in Figure 2. Although the AMJ treatment tended to decrease *LINE-1* methylation levels, there was no significant difference in the whole study group [after vs. before treatment = 97.93 (93.58–99.84)% vs. 98.16 (93.58–99.91)%; P (Wilcoxon matched pairs test) = 0.14] (Figure 2A). We found a significant decrease in *LINE-1* methylation after the AMJ treatment in women (after vs. before treatment = 97.54 ± 1.50 vs. 98.39 ± 0.86%; P (*t*-test for dependent samples) = 0.01) (Figure 2C).

In women, where we demonstrated that polyphenol-rich AMJ treatment significantly changed *LINE-1* methylation, the multiple regression model showed significant effects of average daily folate intake, *MTHFR* C677T genotypes (model CT+TT vs. CC), change (Δ, after vs. before treatment) in triacylglycerols, and change (Δ) in LDL-C on the change (Δ) in *LINE-1* methylation levels, while controlling for age (*R* = 0.72, *R*^2^ = 0.52, adjusted *R*^2^ = 0.36, *P* = 0.03) (Table 2).

**Table 2:** Multiple regression analysis for the change (Δ) in *LINE-1* methylation levels (%) in women who consumed polyphenol-rich *Aronia melanocarpa* juice (AMJ treatment, *N* = 22).

**Predictor variable**	**β**	**Std. error of β**	***P***
Age (years)	−0.40	0.21	0.08
Average daily folate intake (μg)	0.49	0.19	**0.02**
*MTHFR* C677T genotypes (model CT + TT vs. CC)	0.50	0.19	**0.02**
Δ Triacylglycerols (mmol/l)	−0.53	0.20	**0.02**
Δ Low-density lipoprotein cholesterol (mmol/l)	0.46	0.19	**0.03**

*Regression summary: R = 0.72, R^2^ = 0.52, adjusted R^2^ = 0.36, P = 0.03.*

*Δ: change, after treatment value—before treatment value; significant difference at P < 0.05 (bolded text)*.

### Impact of Polyphenol-Rich AMJ Treatment on the Profile of Plasma Fatty Acids

Composition of plasma phospholipids fatty acids was analyzed in women who consumed polyphenol-rich aronia juice, as, only in these subjects, aronia juice consumption was associated with a significant change in *LINE-1* methylation levels. Profiled fatty acids are shown for 15 women treated with aronia juice (Supplementary Table 3), whose plasma samples were available for the analysis, representing a part of previously published data (23). Overall baseline plasma phospholipid fatty acids comprised ~48% SFAs, ~11% MUFAs, and ~41% PUFAs. Among the most abundant fatty acids were palmitic acid (~30.5%) > linoleic acid (~23%) > stearic acid (~17.5%) > arachidonic acid (AA) (~11%), while least quantitatively represented fatty acids were eicosapentaenoic acid (EPA) and adrenic acid (~0.3–0.4%). Within PUFAs, the proportion of omega-6 was ~10-fold higher than that of omega-3 (~37.4 vs. ~3.6%). Comparisons of fatty acid levels after vs. before the AMJ treatment in female subjects revealed a significant decrease in AA/EPA ratio (29.17 ± 15.21 vs. 38.42 (25.96–89.58), respectively; Wilcoxon matched pairs test, *P* = 0.02), as AA levels significantly decreased (10.18 ± 2.16 vs. 11.16 ± 2.61 %, respectively; *t*-test for dependent samples, *P* = 0.01) and EPA levels significantly increased (0.43 ± 0.20 vs. 0.28 ± 0.12 %, respectively; *t*-test for dependent samples, *P* = 0.04) (Supplementary Table 3). Along with AA, dihomo-γ linolenic acid also decreased following the treatment in women [2.34 (1.59–4.33)% vs. 2.89 ± 1.04%, respectively; Wilcoxon matched pairs test, *P* = 0.02]. The same type of change was observed for adrenic acid [0.34 ± 0.12% vs. 0.40 (0.23–0.98)%, respectively; Wilcoxon matched pairs test, *P* = 0.003] (Supplementary Table 3).

The changes (Δ, after vs. before treatment) in levels of individual fatty acids did not significantly correlate with a change (Δ) in *LINE-1* methylation levels, with the exception of adrenic acid (*R* = 0.60, *R*^2^ = 0.36, adjusted *R*^2^ = 0.31, *P* = 0.02). Multiple regression models revealed no significant relations between a change (Δ) in *LINE-1* methylation levels and changes (Δ) in levels of each class of fatty acids: SFAs (*R* = 0.53, *R*^2^ = 0.28, adjusted *R*^2^ = 0.17, *P* = 0.13), MUFAs (*R* = 0.43, *R*^2^ = 0.18, adjusted *R*^2^ = −0.04, P = 0.51), and PUFAs (*R* = 0.86, *R*^2^ = 0.74, adjusted *R*^2^ = 0.49, *P* = 0.09).

## Discussion

The main finding of this study is that the 4-week daily consumption of *A. melanocarpa* juice decreased the *LINE-1* methylation levels in peripheral blood leukocytes in women with CVD risk factors, including overweight and dyslipidemia. Daily treatment with Aronia melanocarpa juice, assigned as AMJ treatment, contained 1.18 g of total polyphenols. This amount corresponds to an estimated average daily intake of about 1 g of total polyphenols from the dietary sources (31), provided that, in the composition of aronia polyphenols, the most abundant are anthocyanins, proanthocyanidins, hydroxycinnamic acids, and flavonols (32), which is in compliance with the findings of a previous study that characterized the composition of aronia juice used in the current research (24). Confirmation that the currently demonstrated significant reduction in *LINE-1* methylation is due to the impact of polyphenols, rather than of other bioactive components of aronia juice, lies in the fact that placebo treatment exerted no significant effects on *LINE-1* methylation. In line with this study, global DNA methylation levels in peripheral leukocytes were significantly reduced after 2-week-long consumption of polyphenol-rich cocoa product, in individuals at cardiovascular risk (13).

Herein observed decreased DNA methylation levels after the consumption of polyphenol-rich aronia juice may be attributed to the role of polyphenols as natural DNMT inhibitors (22). Namely, catechins and their metabolites, which belong to one of the main aronia polyphenol classes—proanthocyanidins (32), can increase the production of S-adenosyl-L-homocysteine (SAH), a potent inhibitor of DNMTs and a participant of the folate-methionine cycle, through the inhibition of catechol-O-methyltransferase (COMT)-mediated O-methylation of catechol substrates (33, 34). Among the main endogenous substrates for both liver and leukocyte, COMT is a catechol estrogen (33, 35), potentially explaining the finding of the present methylation analysis exclusively in women. In addition, catechins suppress MTHFR activity, hence blocking the folate-methionine cycle and reducing the methyl group bioavailability (36). The antioxidant and anti-inflammatory activities of polyphenol-rich aronia products, by which they contribute to cardiovascular protection (17, 37), might be attributed to the role of polyphenols as DNMT inhibitors. By reducing the activity of DNMTs, the prevalent aronia flavonol, quercetin, decreases the promoter methylation levels and activates the expression of target genes, such as a transcription regulator nuclear factor erythroid 2-related factor 2 (38), which is involved in the anti-inflammatory and antioxidant mechanisms in vascular cells and macrophages (38–40). Moreover, aronia polyphenols may be involved in the inflammatory response by affecting the activity/production of DNMTs *via* cytokines, since it has been demonstrated that aronia anthocyanins affected the production of inflammatory mediators (37), and under the influence of IL-1, there have been changes in DNMT expression and genomic methylation in human cells *in vitro* (41).

With regard to our results, the proposed mechanisms of polyphenol-induced inhibition of DNA methylation (33, 34, 36) would depend on daily folate intake and *MTHFR* C677T gene variant. We found that the change (after vs. before the AMJ treatment) in *LINE-1* methylation levels in women correlated directly with average daily folate intake, and this is consistent with previously defined effects of folate on the modulation of DNA methylation (4). Concerning the effects of *MTHFR* C677T gene variant, our finding is in line with the study of Nojima et al. (42), which showed that the global methylation levels in peripheral blood leukocytes were significantly increased in carriers of the 677T allele. This was obtained when the *MTHFR* variant was analyzed in interaction with another factor that affected the methylation of DNA—an inflammatory marker blood concentration (42). Similarly, except with folate intake, our regression model denoted an interaction of the *MTHFR* variant with circulating LDL-C and triacylglycerol levels, as metabolic factors have been associated with *LINE-1* methylation (9, 10, 43). Namely, the current change in *LINE-1* methylation in women correlated positively with the change in LDL-C and negatively with the change in triacylglycerols, which should be pointed out along with the fact that our female subjects are dyslipidemic. So far, few studies have investigated the relationship between *LINE-1* methylation and lipid profile and yielded conflicting results (9, 10, 43), emphasizing the need for further research. The present positive correlation of peripheral blood leukocyte *LINE-1* methylation with circulating LDL-C levels is in line with two studies, which have investigated middle-aged subjects with cardiovascular risk factors and no evidence of CVD (10, 43). Since we have established this positive correlation between changes in *LINE-1* methylation and circulating LDL-C levels in subjects whose *LINE-1* methylation was significantly decreased, following the consumption of aronia juice, our finding may support the currently proposed cardioprotective action of polyphenol-rich aronia products attributed to the role of polyphenols as DNMT inhibitors. Accordingly, the same type of correlation would be expected between changes in *LINE-1* methylation and serum triacylglycerols levels, but we found them to correlate inversely. Still, findings regarding the link of *LINE-1* methylation with serum triacylglycerols are rather inconsistent, as one of the mentioned studies reported a positive correlation (10), while in the other there was no significant relation (43). With respect to pathogenesis of CVD and the proposed explanation, LDL-C was shown to cause vascular endothelial dysfunction in part by changing the DNMT activity and thus altering the DNA methylation, given that, in endothelial cells, LDL-C inhibited the transcription of a gene important in maintaining the endothelial function, *KLF2*, through the activation of DNMT1 (44).

Along with the investigated classical lipid parameters (circulating cholesterol and triacylglycerols), we examined plasma fatty acids profile in women who consumed polyphenol-rich aronia juice, since the treatment significantly changed the *LINE-1* methylation levels only in these individuals, and this change in *LINE-1* methylation was found to correlate with the changes in lipid parameters. We observed a significant reduction of the AA/EPA ratio due to a significant decrease in plasma AA and a significant increase in EPA levels, in target dyslipidemic female subjects who underwent AMJ treatment. Similarly, in a larger group of study participants at cardiovascular risk, from which our substudy group was sorted out, there had also been a significant AA/EPA reduction in aronia polyphenols consumers, noting that this reduction had been due to a significant decrease in AA, while EPA had not increased significantly (23). Furthermore, in a recent study that has investigated the link between *FADS2* gene variants, fatty acid metabolism, and aronia polyphenol intake in the overweight, there was a trend of AA/EPA reduction in individuals who consumed aronia juice, compared to placebo consumers (45). There has been evidence for an association between total plasma AA and ischemic stroke (46), and the increase in circulating EPA levels has been shown to have beneficial effects on cardiovascular health (47). Both findings (46, 47) are expected because the ratio of AA to EPA reflects regulatory mechanisms of the inflammatory process, as these two PUFAs compete for the conversion to two classes of bioactive eicosanoids: pro- and anti-inflammatory (48, 49). Hence, in addition to the well-known markers, such as circulating total cholesterol, LDL-C, and triacylglycerols, the AA/EPA ratio has recently been identified as a sensitive marker of cardiovascular risk, given that a lower AA/EPA ratio was associated with a decreased risk of coronary artery disease, acute coronary syndrome, myocardial infarction, stroke, chronic heart failure, and peripheral artery disease [reviewed in Davinelli et al. (50)]. Our female study subjects who consumed aronia juice had high baseline AA/EPA levels, indicating a chronic low-grade inflammation and an increased risk of cardiovascular events. Thus, a significant decrease in AA/EPA ratio following the AMJ treatment is in line with proposed anti-inflammatory and cardioprotective effects of aronia juice polyphenols, also supporting a suggested relation of these effects with the currently observed decrease in *LINE-1* methylation levels in women.

The findings of the present study should be interpreted in light of limiting factors, primarily a sample size. Nevertheless, the study design has strength regarding the measurement of *LINE-1* methylation levels both before and after each applied treatment, together with the corresponding anthropometric and metabolic parameters, allowing the accurate determination of treatment-dependent changes in parameter values. Another strength of the study is the use of a reliable food frequency questionnaire for the baseline dietary intake assessment with the subsequent analysis of data collected from 24-h dietary recalls, performed by using a validated nutritional platform for comprehensive diet evaluation. This allowed balancing of baseline dietary intake of the study subjects across each of the two interventional treatments, assuming that dietary intake did not change within the 4-week treatment period. Still, the possible confounding effects of temporal changes in dietary habits may not be excluded and represent a limiting factor of the study design. Another limiting factor is a possible interindividual variability in response to polyphenol treatment, related to polyphenol bioavailability and other factors that fell beyond the scope of this study.

In conclusion, the main novelty brought by the present study is a change in *LINE-1* methylation levels after the 4-week habitual consumption of polyphenol-rich aronia juice, which indicates an impact of aronia polyphenols on DNA methylation. The current results suggest cardioprotective effects of aronia polyphenols in subjects at CVD risk, achieved through the modifications of *LINE-1* DNA methylation pattern and AA/EPA ratio. Our findings merit further investigation, particularly in view of the fact that the precision nutrition-driven modulations of both DNA methylation and PUFA profile may become pertinent targets for new approaches in the prevention and treatment of CVD.

## Data Availability Statement

The original contributions presented in the study are included in the article/Supplementary Material, further inquiries can be directed to the corresponding author/s.

## Ethics Statement

The studies involving human participants were reviewed and approved by Ethics Committee of the Clinical Hospital Centre Zemun, Belgrade, Serbia. The patients/participants provided their written informed consent to participate in this study.

## Author Contributions

LS performed the epigenetic, genetic and statistical analyses, interpreted the results, and wrote the manuscript. MZe was involved in the assessments of dietary intake, anthropometric and metabolic parameters, and the analysis of plasma fatty acids composition. AS, MZi, MZe, and LS designed the study. MZe, MZi, and AS revised the manuscript. MBu assisted with the epigenetic analysis. MBo was involved in laboratory work and sample collection. MG contributed to the conceptualization of the study. All authors read and agreed to the final version of the manuscript.

## Conflict of Interest

The authors declare that the research was conducted in the absence of any commercial or financial relationships that could be construed as a potential conflict of interest.

## Abbreviations

AA: arachidonic acid
BMI: body mass index
COMT: catechol-O-methyltransferase
CVD: cardiovascular disease
DBP: diastolic blood pressure
DNMTs: DNA methyltransferases
EDTA: ethylenediaminetetraacetic acid
EPA: eicosapentaenoic acid
Glu: glucose
HDL-C: high-density lipoprotein cholesterol
LDL-C: low-density lipoprotein cholesterol
*LINE-1*: *Long Interspersed Nucleotide Element-1*
MTHFR: methylenetetrahydrofolate reductase
MUFA: monounsaturated fatty acid
PCR: polymerase chain reaction
PUFA: polyunsaturated fatty acid
SAH: S-adenosyl-L-homocysteine
SAM: S-adenosyl-methionine
SBP: systolic blood pressure
SFA: saturated fatty acid
TAG: triacylglycerols
TC: total cholesterol
WC: waist circumference.
