# Supplementary material for: Polyphenol-Rich Aronia melanocarpa Juice Consumption Affects LINE-1 DNA Methylation in Peripheral Blood Leukocytes in Dyslipidemic Women
Source: Front Nutr. 2021 Jun 17;8:689055. doi: 10.3389/fnut.2021.689055 (PMC8247759; doi:10.3389/fnut.2021.689055)
Supplement: Supplementary file 1 [file Table_1.DOC]

**Supplemental Table 1.** Baseline characteristics of the study participants, with respect to gender.

|  | **AMJ**  **women** | **PLB**  **women** | ***P*** | **AMJ**  **men** | **PLB**  **men** | ***P*** |
| --- | --- | --- | --- | --- | --- | --- |
| **No. of subjects** | 22 | 10 |  | 12 | 10 |  |
| **Age (years)** | 42.5 ± 7.2 | 39.0 ± 7.0 | 0.20 | 38.6 ± 4.7 | 38.0 ± 6.9 | 0.82 |
| **Daily**  **energy intake (kCal)** | 1859 (1295–3700) ^#^ | 1616 ± 281 | 0.08 | 2341 ± 544 | 2300 ± 708 | 0.88 |
| **Daily**  **folate intake (μg)** | 226.7 ± 60.0 | 206.0 ± 36.4 | 0.32 | 262.5 ± 95.0 | 272.5 ± 134.2 | 0.84 |
| **SBP (mmHg)** | 117.1 ± 13.4 | 112.0 ± 14.9 | 0.35 | 120.7 ± 13.7 | 129.2 ± 12.9 | 0.15 |
| **DBP (mmHg)** | 72.5 ± 9.8 | 70.9 ± 11.5 | 0.70 | 74.6 ± 10.6 | 71.5 (63.5–103.0) ^#^ | 1.00 |
| **Waist circumference (cm)** | 86.5 (70.0–110.0) ^#^ | 88.2 ± 19.4 | 0.78 | 97.2 ± 8.6 | 96.2 ± 10.5 | 0.80 |
| **BMI (kg/m^2^)** | 26.9 ± 3.6 | 28.2 ± 8.2 | 0.66 | 28.2 ± 3.2 | 27.5 ± 3.8 | 0.63 |
| **Glucose (mmol/l)** | 4.8 (3.8–7.2) ^#^ | 5.1 ± 0.6 | 0.43 | 5.0 ± 0.7 | 5.2 ± 1.0 | 0.63 |
| **TAG (mmol/l)** | 0.8 (0.4–2.0) ^#^ | 1.0 ± 0.4 | 0.79 | 1.8 ± 1.1 | 1.0 (0.5–5.0) ^#^ | 0.17 |
| **TC (mmol/l)** | 5.3 ± 1.1 | 5.5 ± 0.9 | 0.67 | 5.9 ± 1.0 | 4.9 ± 1.2 | **0.04** |
| **HDL-C (mmol/l)** | 1.8 (1.0–2.9) ^#^ | 1.9 ± 0.4 | 0.17 | 1.3 ± 0.3 | 1.4 ± 0.3 | 0.47 |
| **LDL-C (mmol/l)** | 3.3 ± 1.0 | 3.4 ± 0.9 | 0.85 | 3.7 ± 0.9 | 3.2 ± 1.2 | 0.28 |
| ^†^ ***MTHFR* C677T genotype,**  **No. (%)** |  |  |  |  |  |  |
| **CC** | 7 (31.8) | 3 (30.0) |  | 8 (66.7) | 4 (40.0) |  |
| **CT + TT** | 15 (68.2) | 7 (70.0) | 1.00 | 4 (33.3) | 6 (60.0) | 0.39 |

Continuous variables with a normal distribution are presented as mean ± standard deviation; ^#^ continuous variables with a non-normal distribution are presented as median (minimum–maximum); *P* - values related to between-treatment difference in the variable distribution within the group (women or men), bolded text denotes significant difference (*P* < 0.05).

^†^ The observed *MTHFR* C677T genotype frequencies are consistent with Hardy-Weinberg equilibrium, in each analysed group (χ^2^ test, *P* > 0.05).

AMJ - polyphenol-rich *Aronia melanocarpa* juice treatment; PLB - polyphenol-free beverage, placebo treatment; SBP - systolic blood pressure; DBP - diastolic blood pressure; BMI - body mass index; TAG - triacylglycerols; TC - total cholesterol; HDL-C - high-density lipoprotein cholesterol; LDL-C - low-density lipoprotein cholesterol.
