# Supplementary material for: Polyphenol-Rich Aronia melanocarpa Juice Consumption Affects LINE-1 DNA Methylation in Peripheral Blood Leukocytes in Dyslipidemic Women
Source: Front Nutr. 2021 Jun 17;8:689055. doi: 10.3389/fnut.2021.689055 (PMC8247759; doi:10.3389/fnut.2021.689055)
Supplement: Supplementary file 2 [file Table_2.DOC]

**Supplemental Table 2.** Effects of polyphenol-rich *Aronia melanocarpa* juice consumption on anthropometric and metabolic parameters in the whole study group, and separated by gender.

|  | **AMJ**  **all** | | ***P*** | **AMJ**  **women** | ***P*** | **AMJ**  **men** | | ***P*** | **PLB**  **all** | ***P*** | | **PLB**  **women** | ***P*** | | | **PLB**  **men** | ***P*** |
| --- | --- | --- | --- | --- | --- | --- | --- | --- | --- | --- | --- | --- | --- | --- | --- | --- | --- |
|  | (N = 34) | |  | (N = 22) |  | (N = 12) | |  | (N = 20) | |  | (N = 10) |  | | | (N = 10) |  |
|  |  |  |  |  |  |  |  |  |  |  |  |  |  |  |  |  |  |
| **SBP before treatment (mmHg)** | 118.3 ± 13.4 | |  | 117.1 ± 13.4 |  | 120.7 ± 13.7 | |  | 120.6 ± 16.2 | |  | 112.0 ± 14.9 |  | | | 129.2 ± 12.9 |  |
| **SBP after treatment (mmHg)** | 115.2 ± 13.5 | | 0.13 | 111.9 ± 13.6 | 0.09 | 121.1 ± 11.4 | | 0.87 | 119.0 ± 16.3 | | 0.35 | 110.2 ± 14.0 | 0.31 | | | 127.8 ± 13.9 | 0.65 |
| **DBP before treatment (mmHg)** | 73.2 ± 10.0 | |  | 72.5 ± 9.8 |  | 74.6 ± 10.6 | |  | 74.0 ± 13.7 | |  | 70.9 ± 11.5 |  | | | 71.5 (63.5–103)^#^ |  |
| **DBP after treatment (mmHg)** | 70.2 ± 10.2 | | **0.02** | 69.0 ± 9.7 | 0.06 | 72.3 ± 11.2 | | 0.14 | 72.1 ± 13.3 | | 0.12 | 68.0 ± 11.9 | **0.03** | | | 76.2 ± 14.1 | 0.57 |
| **WC before treatment**  **(cm)** | 89.8 ± 10.9 | |  | 86.5 (70–110)^#^ |  | 97.2 ± 8.6 | |  | 92.2 ± 15.7 | |  | 88.2 ± 19.4 |  | | | 96.2 ± 10.5 |  |
| **WC after treatment**  **(cm)** | 89.4 ± 11.4 | | 0.42 | 84.7 ± 9.8 | 0.12 | 98.0 ± 9.0 | | 0.39 | 91.0 ± 15.9 | | **0.01** | 87.1 ± 19.3 | 0.05 | | | 95.0 ± 11.2 | 0.10 |
| **BMI before treatment (kg/m^2^)** | 27.4 ± 3.5 | |  | 26.9 ± 3.6 |  | 28.2 ± 3.2 | |  | 27.8 ± 6.2 | |  | 28.2 ± 8.2 |  | | | 27.5 ± 3.8 |  |
| **BMI after treatment (kg/m^2^)** | 27.3 ± 3.5 | | 0.32 | 26.8 ± 3.6 | 0.27 | 28.2 ± 3.2 | | 0.95 | 27.7 ± 6.3 | | 0.35 | 28.1 ± 8.2 | 0.73 | | | 27.3 ± 3.9 | 0.38 |
| **Glu before treatment (mmol/l)** | 4.8 (3.8–7.2)^#^ | |  | 4.8 (3.8–7.2)^#^ | 0.51 | 5.0 ± 0.7 | |  | 5.1 ± 0.8 | |  | 5.1 ± 0.6 |  | | | 5.2 ± 1.0 |  |
| **Glu after treatment (mmol/l)** | 4.7 (3.8–7.9)^#^ | | 0.60 | 4.8 ± 0.8 |  | 4.9 (4.0–7.9)^#^ | | 0.69 | 5.0 ± 0.9 | | 0.27 | 4.7 ± 0.8 | | **0.04** | | 5.3 ± 0.9 | 0.27 |
| **TAG before treatment (mmol/l)** | 0.9 (0.4–4.1)^#^ | |  | 0.8 (0.4–2.0)^#^ |  | 1.8 ± 1.1 | |  | 0.9 (0.5–5.0)^#^ | |  | 1.0 ± 0.4 | | |  | 1.0 (0.5–5.0)^#^ |  |
| **TAG after treatment (mmol/l)** | 0.9 (0.4–8.4)^#^ | | 0.98 | 0.8 (0.4–2.7)^#^ | 0.92 | 1.5 (0.8–8.4)^#^ | | 0.84 | 1.2 (0.6–4.5)^#^ | | 0.06 | 1.1 ± 0.5 | | | 0.22 | 1.4 (0.6–4.5)^#^ | 0.14 |
| **TC before treatment (mmol/l)** | 5.5 ± 1.1 | |  | 5.3 ± 1.1 |  | 5.9 ± 1.0 | |  | 5.2 ± 1.0 | |  | 5.5 ± 0.9 | | |  | 4.9 ± 1.2 |  |
| **TC after treatment (mmol/l)** | 5.6 ± 1.2 | | 0.36 | 5.4 ± 1.2 | 0.62 | 6.0 ± 1.1 | | 0.43 | 5.3 ± 0.8 | | 0.52 | 5.4 ± 0.7 | | | 0.87 | 5.1 ± 0.9 | 0.37 |
| **HDL-C before treatment (mmol/l)** | 1.5 (0.8–2.9)^#^ | |  | 1.8 (1.0–2.9)^#^ |  | 1.3 ± 0.3 | |  | 1.6 ± 0.4 | |  | 1.9 ± 0.4 | | |  | 1.4 ± 0.3 |  |
| **HDL-C after treatment (mmol/l)** | 1.5 ± 0.4 | **0.004** | | 1.7 ± 0.4 | 0.12 | 1.2 ± 0.3 | **0.005** | | 1.5 ± 0.4 | **0.04** | | 1.8 ± 0.4 | | | 0.09 | 1.3 ± 0.3 | 0.27 |
| **LDL-C before treatment (mmol/l)** | 3.5 ± 0.9 | |  | 3.3 ± 1.0 |  | 3.7 ± 0.9 | |  | 3.3 ± 1.0 | |  | 3.4 ± 0.9 | | |  | 3.2 ± 1.2 |  |
| **LDL-C after treatment (mmol/l)** | 3.5 ± 1.0 | | 0.98 | 3.3 ± 1.0 | 0.67 | 3.8 ± 1.1 | | 0.63 | 3.4 ± 0.9 | | 0.53 | 3.4 ± 0.8 | | | 0.99 | 3.3 ± 1.0 | 0.44 |

Continuous variables with a normal distribution are presented as mean ± standard deviation; ^#^ continuous variables with a non-normal distribution are presented as median (minimum–maximum); *P* - values related to the within-group difference upon treatment consumption, bolded text denotes significant difference (*P* < 0.05).

N - number of subjects; AMJ - polyphenol-rich *Aronia melanocarpa* juice treatment; PLB - polyphenol-free beverage, placebo treatment; SBP - systolic blood pressure; DBP - diastolic blood pressure; WC - waist circumference; BMI - body mass index; Glu - glucose; TAG - triacylglycerols; TC - total cholesterol; HDL-C - high-density lipoprotein cholesterol; LDL-C - low-density lipoprotein cholesterol.
