# Supplementary material for: Polyphenol-Rich Aronia melanocarpa Juice Consumption Affects LINE-1 DNA Methylation in Peripheral Blood Leukocytes in Dyslipidemic Women
Source: Front Nutr. 2021 Jun 17;8:689055. doi: 10.3389/fnut.2021.689055 (PMC8247759; doi:10.3389/fnut.2021.689055)
Supplement: Supplementary file 3 [file Table_3.DOC]

**Supplemental Table 3.** Composition of plasma phospholipids fatty acids in women (N = 15) who consumed polyphenol-rich *Aronia melanocarpa* juice (AMJ treatment).

|  | **Before treatment** | **After treatment** | ***P*** |
| --- | --- | --- | --- |
| **Palmitic acid, 16:0 (%)** | 30.46 ± 2.93 | 31.30 ± 1.34 | 0.25 |
| **Stearic acid, 18:0 (%)** | 17.52 ± 1.59 | 18.21 ± 1.62 | 0.13 |
| **Palmitoleic acid, 16:1n-7 (%)** | 0.58 ± 0.18 | 0.55 ± 0.25 | 0.72 |
| **Oleic acid, 18:1n-9 (%)** | 7.70 ± 0.64 | 7.70 ± 1.20 | 0.99 |
| **Vaccenic acid, 18:1n-7 (%)** | 2.66 ± 0.41 | 2.78 ± 0.52 | 0.37 |
| **Linoleic acid, 18:2n-6 (%)** | 22.94 ± 3.50 | 22.95 ± 3.04 | 0.99 |
| **Dihomo-γ linolenic acid, 20:3n-6 (%)** | 2.89 ± 1.04 | 2.34 (1.59–4.33) ^#^ | **0.02** |
| **Arachidonic acid, 20:4n-6 (%)** | 11.16 ± 2.61 | 10.18 ± 2.16 | **0.01** |
| **Adrenic acid, 22:4n-6 (%)** | 0.40 (0.23–0.98) ^#^ | 0.34 ± 0.12 | **0.003** |
| **Eicosapentaenoic acid, 20:5n-3 (%)** | 0.28 ± 0.12 | 0.43 ± 0.20 | **0.04** |
| **Docosapentaenoic acid, 22:5n-3 (%)** | 0.50 (0.33–0.69) ^#^ | 0.48 ± 0.19 | 0.22 |
| **Docosahexaenoic acid, 22:6n-3 (%)** | 2.81 ± 0.69 | 2.66 ± 0.66 | 0.40 |
| **Arachidonic/Eicosapentaenoic acid ratio** | 38.42 (25.96–89.58) ^#^ | 29.17 ± 15.21 | **0.02** |
| **Arachidonic/Docosahexaenoic acid ratio** | 4.07 ± 0.77 | 3.99 ± 1.11 | 0.79 |

Levels of fatty acids with a normal distribution are presented as mean ± standard deviation; ^#^ levels of fatty acids with a non-normal distribution are presented as median (minimum–maximum); *P* - values related to the within-group difference upon treatment consumption, bolded text denotes significant difference (*P* < 0.05).
